# Stimulation of the Migration and Expansion of Adult Mouse Neural Stem Cells by the FPR2-Specific Peptide WKYMVm

**DOI:** 10.3390/life11111248

**Published:** 2021-11-17

**Authors:** Yang Woo Kwon, Sungwon Bae, Yeon Suk Jo, Youngsuk Seo, Jong Hyuk Yoon

**Affiliations:** 1 Neurodegenerative Diseases Research Group, Korea Brain Research Institute, Daegu 41062, Korea; rnjsdiddn@kbri.re.kr (Y.W.K.); maria1101@kbri.re.kr (S.B.); jys0801@kbri.re.kr (Y.S.J.); ysseo910@kbri.re.kr (Y.S.); 2 Department of Brain-Cognitive Science, Daegu-Gyeongbuk Institute of Science and Technology (DGIST), Daegu 42988, Korea

**Keywords:** neural stem cells, formyl peptide receptor 2, migration, expansion, WKYMVm

## Abstract

Neural stem cells (NSCs) are multipotent cells capable of self-renewal and differentiation into different nervous system cells. Mouse NSCs (mNSCs) are useful tools for studying neurogenesis and the therapeutic applications of neurodegenerative diseases in mammals. Formyl peptide receptor 2 (FPR2), expressed in the central nervous system and brain, is involved in the migration and differentiation of murine embryonic-derived NSCs. In this study, we explored the effect of FPR2 activation in adult mNSCs using the synthetic peptide Trp-Lys-Tyr-Met-Val-D-Met-NH2 (WKYMVm), an agonist of FPR2. After isolation of NSCs from the subventricular zone of the adult mouse brain, they were cultured in two culture systems—neurospheres or adherent monolayers—to demonstrate the expression of NSC markers and phenotypes. Under different conditions, mNSCs differentiated into neurons and glial cells such as astrocytes, microglia, and oligodendrocytes. Treatment with WKYMVm stimulated the chemotactic migration of mNSCs. Moreover, WKYMVm-treated mNSCs were found to promote proliferation; this result was confirmed by the expansion of mNSCs in Matrigel and the increase in the number of Ki67-positive cells. Incubation of mNSCs with WKYMVm in a supplement-free medium enhanced the survival rate of the mNSCs. Together, these results suggest that WKYMVm-induced activation of FPR2 stimulates cellular responses in adult NSCs.

## 1. Introduction

Neural stem cells (NSCs) are present in the major neurogenic regions of the brain, including the subgranular zone in the dentate gyrus of the hippocampus and the subventricular zone of the lateral ventricle [1,2,3], and can be isolated from the brains of fetuses and adults [4]. Neural stem cells possess stem cell properties such as self-renewal, long-term viability, and potential for differentiation into neural lineage cell types, including neurons and glial cell lineages such as astrocytes and oligodendrocytes [5,6,7]. In addition to their ability to differentiate into multiple cell lineages, NSCs play an important role in the brain by secreting neurotrophic factors that regulate the local immune system, apoptosis, and homeostasis and protect the host cells [8,9,10]. Various studies have demonstrated that transplantation of NSCs improves the therapeutic efficacy in Alzheimer’s disease, Parkinson’s disease, and other neurological disease models [11,12]. These studies suggest that NSCs enhance memory, cognition, behavior, and motor ability in neurodegenerative diseases by stimulating neuronal survival and synaptic function. In addition, it has been demonstrated in a disease model that endogenous NSCs are mobilized to the lesion site and play a crucial role in neuronal regeneration [13,14]. Although NSCs can be a powerful tool for treating neurological diseases, the therapeutic efficacy of NSCs in clinical settings has not been satisfactory, so their application is insufficient.

The formyl peptide receptor (FPR) is a chemoattractant receptor of the G protein-coupled receptor family and is known to play key roles in host defense, inflammation, and neovascularization by regulating the activity of different cells [15,16,17]. The members of three FPRs (FPR1, FPR2, and FPR3) have been identified in humans, of which FPR1 and FPR2 were identified in mice to be homologous to human FPR1 and FPR2 [15,17,18]. Formyl peptide receptors are typically expressed in immune cells such as neutrophils, monocytes, and macrophages [19] and function by expression in diverse cell types such as endothelial cells, fibroblasts, and neural cells [20,21,22]. Formyl peptide receptors can be activated by a variety of agonist ligands [23], and FPR2 is known to be a high-affinity receptor for WKYMVm (Trp-Lys-Tyr-Met-Val-D-Met-NH_2_), a modified synthetic hexapeptide [24,25]. WKYMVm not only activates the infiltration of monocytes and macrophages but also stimulates neutrophils, lymphocytes, and leukocytes by binding to FPR2 in vivo [26,27,28].

The formyl peptide receptor 2 has been detected in the brain and spinal cord, and there is increasing evidence that FPR2 is expressed in the central nervous system and interacts with ligands [29]. It has been reported that FPR2 is expressed in NSCs from the fetal phase. MMK-1, an FPR2 agonist, promotes the migration and differentiation of fetal-derived NSCs, indicating a function of FPR2 in the cellular responses of NSCs [30]. However, the role of FPR2 in adult brain NSCs has not yet been explored.

In the present study, NSCs were isolated from adult mice, and their stem cell characteristics were analyzed. In addition, we investigated the effect of the FPR2 agonist WKYMVm on the migration and expansion of adult mNSCs.

## 2. Materials and Methods

### 2.1. Materials

Dulbecco’s modified Eagle’s medium: Nutrient Mixture F-12 (DMEM/F12), Neurobasal Medium, B-27 supplement, phosphate-buffered saline (PBS) buffer, fetal bovine serum (FBS) and anti-glial fibrillary acidic protein (GFAP) were purchased from Thermo Fisher Scientific (Waltham, MA, USA). Recombinant mouse epidermal growth factor (EGF) and basic fibroblast growth factor (bFGF) were purchased from R&D Systems (Minneapolis, MN, USA). Poly-L-ornithine (PLO), laminin and bovine serum albumin (BSA) were purchased from Sigma-Aldrich (St. Louis, MO, USA). Anti-Nestin and oligodendrocyte marker O4 (O4) antibodies were purchased from Millipore (Burlington, MA, USA). Anti-Sox2 and Ki67 antibodies were purchased from Abcam (Cambridge, UK). Anti-ionized calcium-binding adaptor molecule 1 (Iba1) was purchased from FUJIFILM Wako Chemicals (Richmond, VA, USA). Growth-factor-reduced Matrigel was purchased from BD Biosciences (Bedford, MA, USA). Alexa 568 goat anti-mouse, Alexa 568 goat anti-rat, Alexa 488 goat anti-rabbit, and Alexa 488 and 568 goat anti-rabbit secondary antibodies were purchased from Life Technologies (Carlsbad, CA, USA). WKYMVm (Trp-Lys-Tyr-Met-Val-D-Met-NH_2_) was purchased from Anygen Inc. (Gwangju, Korea).

### 2.2. Isolation of Primary mNSCs and Cell Culture

The mouse NSCs (mNSCs) were isolated from 8-week-old male mouse brains. After collecting the brain, the whole brain was washed with cold PBS buffer; then, the subventricular region was dissected and chopped into small pieces. The chopped tissue was suspended in 1 mL of digestion buffer containing 0.02% papain and 500 μM of ethylenediaminetetraacetic acid (EDTA) and incubated at 37 °C for 30 min. After stopping the enzymatic reaction by adding 1 mL of DMEM/F12 containing a digestion inhibitor, the digested tissue was filtered through a 40 μm cell strainer to remove the debris and non-dissociated tissue. The floating cells were separated by centrifugation at 300× *g* for 5 min. The cell pellet was resuspended in the growth medium, DMEM/F12 supplemented with 2% B-27, 100 U/mL penicillin, and 100 µg/mL streptomycin containing 20 ng/mL EGF and bFGF, after which the cells were plated on uncoated 25 cm^2^ flasks. The mNSCs were maintained at 37 °C in a 5% CO atmosphere and with EGF and bFGF every 2 d. The primary mNSCs were cultured until neurospheres reached approximately 100–200 μm and were defined as passage “0.” For passaging mNSCs, neurospheres were collected by centrifugation and were dissociated into single cells with 0.05% trypsin containing 0.02% EDTA, after which they were maintained by culturing in the growth medium described above. The passage number of mNSCs used in these experiments was between three and five.

### 2.3. Adherent Monolayer Culture and Differentiation of mNSCs

The adherent monolayer culture and differentiation of mNSCs into neural cell types were conducted based on a previously reported protocol [31,32]. For the adherent monolayer culture, the neurospheres were dissociated into single cells with 0.05% trypsin containing 0.02% EDTA at 37 °C for 5 min, and then seeded onto 20 μg/mL PLO and 5 μg/mL laminin (PLO/laminin)-coated cell culture plates with growth medium. After confirming the cells attached to the plate surface, growth factors were supplemented by exchanging the growth medium to fresh medium every 2 to 3 d.

For differentiation of mNSCs, neurospheres were dissociated into single cells, after which the cells were plated onto PLO/laminin-coated 12 mm coverslips at 1 × 10^5^ cells/mL in growth medium. Once the cells were attached to the coverslip, the growth medium was replaced with a differentiation medium such as neurobasal medium or DMEM/F12 containing 10% FBS to remove the growth factors. After 6 d, to determine whether differentiation into various neuronal cell types occurred, the differentiated cells were immunostained using specific marker antibodies for neurons, astrocytes, microglia, or oligodendrocytes.

### 2.4. Immunocytochemistry Analysis

For immunofluorescence image analysis of the mNSCs, the neurospheres or adherent cells were fixed in PBS containing 4% paraformaldehyde for 10 min and washed three times with PBS. Then, the neurospheres or adherent cells were permeabilized with PBS containing 0.2% Triton X-100 for 10 min and blocked with PBS containing 5% BSA for 30 min. All procedures were performed at room temperature.

The neurospheres were stained with mouse anti-Nestin or rabbit anti-Sox2 antibodies. The specimens were incubated with Alexa 568 goat anti-mouse or Alexa 488 goat anti-rabbit secondary antibodies. Cells differentiated from the mNSCs were stained with primary antibodies such as rabbit anti-neuronal nuclei (NeuN), rat anti-GFAP, rabbit ant-Iba1, or mouse anti-O4. The primary antibodies were detected using Alexa 488 or 586 goat anti-rabbit, Alexa 568 goat anti-rat, or Alexa 568 goat anti-mouse secondary antibodies. The specimens were finally washed and mounted in Vectashield medium (Vector Laboratories, San Francisco, CA, USA) containing 4′,6-diamidino-2-phenylindole (DAPI) to visualize nuclei. The stained neurospheres and cells were visualized using a laser scanning confocal microscope (Nikon, A1R-MP, Nikon Corporation, Tokyo, Japan) under the high-power field (×200).

### 2.5. Western Blotting

The cells were washed twice with PBS, and then lysed in lysis buffer (1% Triton X-100, 50 mM (pH 7.4) Tris, 2 mM CaCl_2_, and 2 mM MgCl_2_, 1 mM EDTA, 1 mM phenylmethylsulfonyl fluoride (PMSF), 1 mg/L pepstatin, 1 mg/L leupeptin, and 2 mg/L aprotinin). The cell lysates were centrifuged at 14,000 rpm for 25 min at 4 °C to pellet the insoluble materials. The cell lysates were resolved by sodium dodecyl sulfate-polyacrylamide gel electrophoresis (SDS-PAGE), transferred onto nitrocellulose membranes, and then stained with 0.1% Ponceau S solution (Sigma-Aldrich) to ensure equal loading of the samples. After blocking with 5% non-fat milk for 30 min, the membranes were incubated with primary antibodies overnight at 4 °C, and the bound antibodies were visualized with horseradish peroxidase (HRP)-conjugated secondary antibodies using an enhanced chemiluminescence Western blotting system (Lumigen ECL Ultra, Southfield, MI, USA).

The quantification of band intensities was conducted using the Image J software (ver. 1.53k). The protein levels were normalized to those of actin using the ratio of the intensity of individual bands to the intensity of the actin band. Cell lysis from mouse NSCs or cultured primary neurons were subjected to immunoblot analyses with the indicated antibodies. Actin-specific antibody was used for normalization.

### 2.6. Cell Migration Assay

Mouse NSC migration was assayed using a disposable 96-well chemotaxis chamber (Neuro Probe, Inc., Gaithersburg, MD, USA). The neurospheres were harvested and dissociated with 0.05% trypsin containing 0.02% EDTA into single cells, washed once, and suspended in DMEM/F12 at a 1 × 10^4^ cells/mL concentration. A membrane filter with 8 μm pores for the chemotaxis chamber was pre-coated overnight with 20 μg/mL rat-tail collagen at 4 °C. An aliquot (35 μL) of mNSC suspension was loaded into the upper chamber, and WKYMVm or EGF was then placed in the lower chamber. After incubating the cells for 12 h at 37 °C and 5% CO_2_, the filters were disassembled, and the upper surface of each filter was scraped free of cells by wiping with a cotton swab. The number of cells that migrated to the lower surface of each filter was determined by counting the cells in five random locations under a microscope at ×100 magnification after staining with Hoechst 33342.

### 2.7. Matrigel Assay

To assess the expansion of the NSCs, growth-factor-reduced Matrigel was added to 96-well culture plates and polymerized for 30 min at 37 °C. Mouse NSCs (1 × 10^4^) were seeded on Matrigel-coated plates and cultured in DMEM/F12 medium supplemented with 2% B-27, followed by treatment with WKYMVm or EGF. After incubating the cells at 37 °C and 5% CO_2_ for 24 h, the expansion rate was photographed with a digital camera in four random microscopic fields and quantified by measuring the length using Image J software (version 1.50i).

### 2.8. Cell Proliferation Assay

The effects of WKYMVm on mNSC proliferation were investigated by immunocytochemistry. Mouse NSCs were seeded in PLO/laminin-coated 24-well culture plates and cultured with DMEM/F12 containing 2% B-27, supplemented with WKYMVm or EGF for 24 h. The cells were fixed in PBS containing 4% paraformaldehyde for 10 min, permeabilized with PBS containing 0.2% Triton X-100 for 10 min, and blocked with PBS containing 5% bovine serum albumin. Specimens were incubated with rabbit anti-Ki67 antibody for 2 h and Alexa 488 goat anti-rabbit secondary antibodies for 1 h. The specimens were finally washed and mounted in Vectashield medium with DAPI, and images of the specimen were collected using a laser scanning confocal microscope.

### 2.9. Cell Survival Assay

The effects of WKYMVm on the survival of mNSCs were investigated using the Cell Counting Kit-8 (CCK-8) assay kit (Dojindo, Rockville, MD, USA). The mNSCs were seeded in with PLO/laminin-coated 24-well culture plates at approximately 5 × 10^4^ cells per well and incubated in DMEM/F12 without B-27 supplementation, followed by treatment with or without WKYMVm for 1, 3, and 5 d. WKYMVm was added to each plate once every 2 d. The CCK-8 solution was added on days 1, 3, and 5 to each well of the plate, followed by incubation for 2 h. Cell viability was determined by measuring the absorbance at 450 and 630 nm wavelength using a microplate reader. All measurements were conducted in triplicates.

### 2.10. Statistical Analysis

The results of the multiple observations were presented as mean ± standard deviation (SD). For the multivariate data analysis, group differences were assessed using one- or two-way analysis of variance (ANOVA), followed by Scheffé’s post hoc test.

## 3. Results

### 3.1. Isolation of Adult NSCs from the Mouse Subventricular Zone

**Figure 1:**
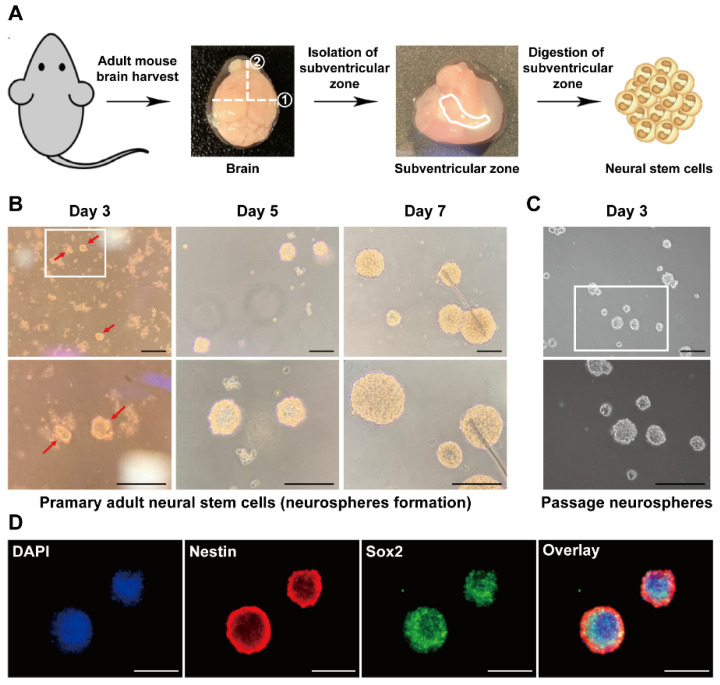
Isolation of adult neural stem cells (NSCs) from mouse subventricular zone and formation of the neurospheres. (**A**) Overview of the isolation protocol for adult NSCs (mNSCs). Brains were harvested from 8-week-old mice, and the subventricular zone was dissected. The dissected subventricular zone was completely minced into small pieces, and the cells were isolated by suspending the minced tissue in digestion buffer. For the neurosphere culture of the isolated cells, the cells were cultured in an NSC growth medium containing growth factors. (**B**) Representative images of the primary mNSC neurosphere formation were obtained by culturing mNSCs. After 3 d of in vitro culture, the primary neurospheres were observed, and the neurospheres grew over time. Red arrows indicate primary neurospheres. (**C**) After 7 d of culture, the primary neurospheres were dissociated into single cells and passaged. The cultured single mNSCs formed neurospheres and were maintained passages. (**D**) The neurospheres were identified by immunofluorescence staining with antibodies against Nestin (red) and Sox2 (green) on day 5 after culture. The nuclei were counterstained with 4′,6-diamidino-2-phenylindole (DAPI) (blue). Scale bar = 200 µm.

Neural stem cells reside in several brain regions and can be isolated from the subventricular and subgranular zones. Neural stem cells, which play an important role in brain function, can be isolated from the adult brain, and a culture system to form neurospheres has been established to obtain NSCs in vitro [33]. Cells were isolated by dissecting and digesting the subventricular zone from the mouse brain (Figure 1A) to isolate adult mouse NSCs (mNSCs. When the isolated cells were sphere-cultured in an NSC growth medium containing growth factors, we tested whether multipotent neurospheres, a characteristic of mNSCs, were formed. As shown in Figure 1B, the cultured cells formed neurospheres, and neurosphere growth was observed time-dependently. We next investigated whether the passage of mNSCs that form neurospheres was maintained. After dissociating primary neurospheres into single cells, the dissociated mNSCs formed neurospheres due to sphere culture (Figure 1C). When the phenotypes of the mNSCs were confirmed by immunostaining with antibodies against NSC-specific markers such as Nestin and Sox2, both Nestin and Sox2 were expressed in neurospheres (Figure 1D). These results suggest that the cells isolated from the subventricular zone of adults are mNSCs.

### 3.2. Characterization of Adult-Derived mNSCs

**Figure 2:**
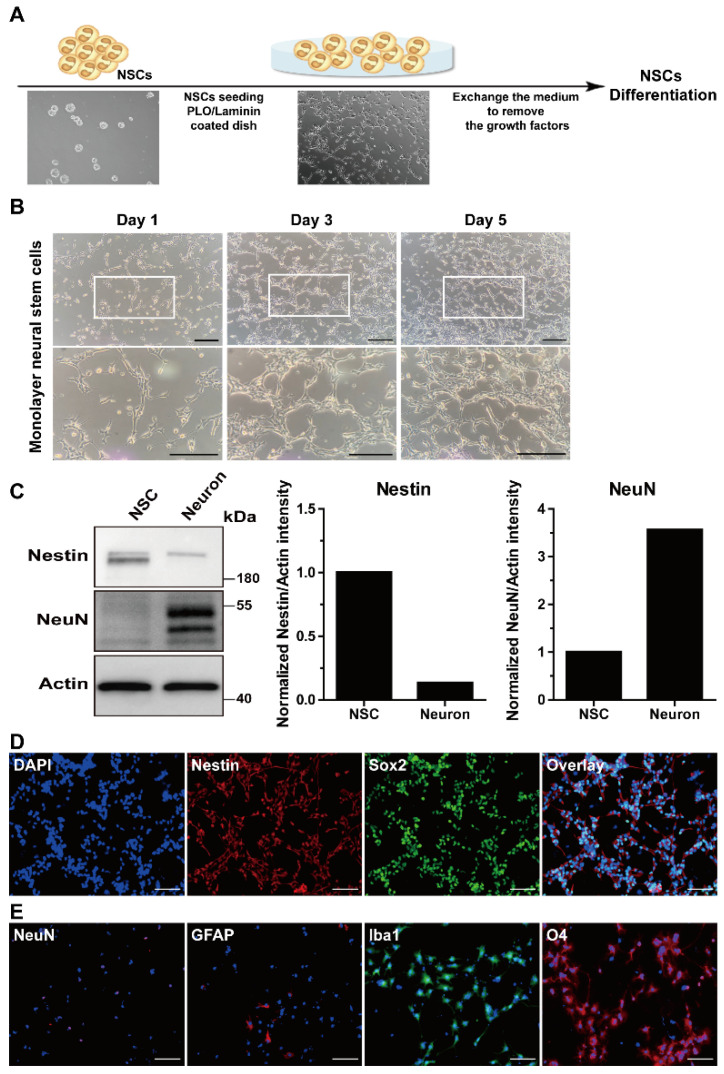
Adherent monolayer culture and characterization of subventricular-zone-derived adult-derived mNSCs. (**A**) Overview of the adherent monolayer culture and characterization analysis protocol for adult mNSCs. The collected neurospheres dissociated into single cells were seeded on poly-L-ornithine (PLO)/laminin-coated plates for monolayer culture, followed by incubation in the growth medium with growth factors. For adherent mNSCs, growth and specific markers were identified, and differentiation into brain cell types was induced in a differentiation medium without growth factors. (**B**) The single mNSCs were attached to the PLO/laminin-coated plates after 1 d, which proliferated. The number of adherent mNSCs increased 5 d after maintenance. Scale bar = 200 µm. (**C**) Western blot analysis of mNSCs and primary neurons using NSC marker (Nestin), neuron marker (neuronal nuclei, NeuN), and actin is shown. Quantitative Western blotting data of Nestin and NeuN using densitometry for actin in mNSCs and primary neurons. (**D**) Representative images for immunocytochemistry of the adherent mNSCs after monolayer culture. Fluorescence images of the adherent mNSCs after immunolabeling with DAPI (blue), Nestin (red), and Sox2 (green) are shown. Scale bar = 100 µm. (**E**) The mNSCs, on day 6 after induced differentiation, were identified using immunofluorescence staining with NeuN (green), glial fibrillary acidic protein (GFAP) (red), ionized calcium-binding adaptor molecule 1 (Iba1) (green), and oligodendrocyte marker O4 (O4) (red) antibodies. The nuclei were counterstained with DAPI (blue), and the merged images are shown. Scale bar = 100 µm.

Neural stem cells can self-renew and differentiate into various types of brain cells such as neurons, astrocytes, and oligodendrocytes [34]. Dissociated single cells from the neurospheres were seeded on PLO/laminin-coated plates for adherent monolayer culture and were cultured in mNSC growth medium (Figure 2A) to determine the characteristics of the adult mNSCs. As shown in Figure 2B, monolayer-cultured mNSCs continuously exhibited proliferation. The levels of the mNSC markers were determined using Western blotting. The Nestin levels were higher in the mNSCs than those in the mouse primary neurons, whereas the expression of NeuN, a neuron-specific marker, was not detected in the mNSCs (Figure 2C). Images of the uncropped original Western blot are provided in Supplementary Figure S1. To further verify the mNSC phenotypes, we measured the expression of Nestin and Sox2 by immunostaining. Monolayer-cultured mNSCs showed the expression of Nestin and Sox2 (Figure 2D).

Proliferating mNSCs were plated on PLO/laminin-coated coverslips with a growth medium containing growth factors to investigate whether mNSCs isolated from the adult mouse subventricular zone can differentiate into brain cell types. After 2 d, the cells were attached to the coverslips, the growth medium was exchanged with differentiation medium to remove growth factors, and the cells were cultured for 6 d. The cells that differentiated from the mNSCs expressed the neuronal marker NeuN, the astrocyte marker GFAP, the microglia marker Iba1, and the oligodendrocyte marker O4, indicating that the mNSCs differentiated into different brain cell types (Figure 2E). These results suggest that cells isolated from the adult mouse subventricular zone exhibit the phenotypes and characteristics of mNSCs.

### 3.3. WKYMVm Stimulates Migration of Adult mNSC

**Figure 3:**
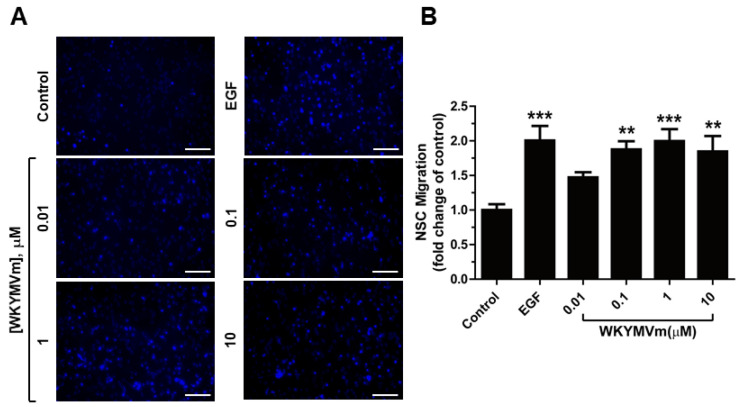
Effects of WKYMVm (Trp-Lys-Tyr-Met-Val-D-Met-NH_2_) on the migration activities of mNSCs. (**A**) Representative images of migration in response to epidermal growth factor (EGF, 10 ng/mL) or various concentrations of WKYMVm are shown (left panels). Scale bar = 100 µm. (**B**) Migration of mNSCs was measured using a chemotaxis chamber in response to EGF or various concentrations of WKYMVm after 12 h of incubation. Data indicate mean ± standard deviation (SD). ** *p* < 0.01 and *** *p* < 0.001 versus control (*n* = 9).

According to a report, FPR2 is involved in rat fetus-derived NSC migration [30]. We tested the effect of WKYMVm on the migration capacity of mNSCs using a chamber migration assay to evaluate whether WKYMVm induces the chemotactic capacity of mNSCs. As shown in Figure 3A,B, WKYMVm promoted the migration of NSCs, with a maximal effect at 1 µM (to a level comparable to that of EGF). These results suggest that WKYMVm acts as a chemoattractant for mNSCs.

### 3.4. WKYMVm Increases the Expansion and Viability of Adult mNSCs

**Figure 4:**
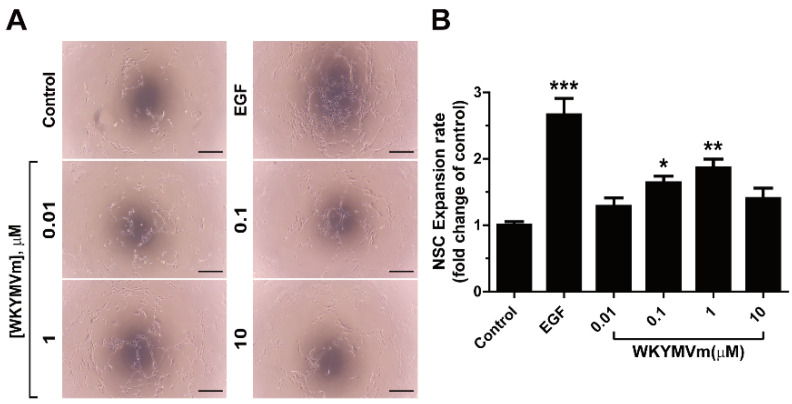
Effects of WKYMVm on the expansion of mNSCs in Matrigel-coated plates. (**A**) Representative images of mNSC expansion in response to EGF (10 ng/mL) or various concentrations of WKYMVm in Matrigel-coated plates are shown. Scale bar = 200 µm. (**B**) Expansion of mNSCs was quantified by measuring the length of the connection between cells in response to EGF or various concentrations of WKYMVm after 24 h of incubation. Data indicate mean ± SD. * *p* < 0.05, ** *p* < 0.01, and *** *p* < 0.001 versus control (*n* = 12).

mNSCs separated from the neurospheres were seeded onto Matrigel-coated plates to investigate whether WKYMVm enhances the expansion ability of mNSCs. When the effect of WKYMVm on mNSC expansion was measured in Matrigel, WKYMVm treatment increased the mNSC expansion (Figure 4A,B).

**Figure 5:**
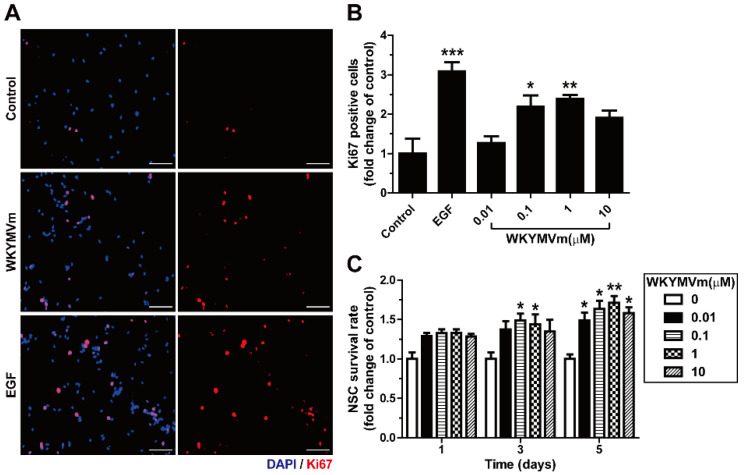
Effects of WKYMVm on the proliferation and survival of mNSCs. (**A**) Representative images of proliferation in response to WKYMVm (0.1 µM) or EGF (10 ng/mL) are shown (left panel). The proliferative effect of WKYMVm on the mNSCs was measured by staining with anti-Ki67 antibody (red). Nuclei were counterstained with DAPI (blue). Scale bar = 100 µm. (**B**) Numbers of Ki67-positive nuclei per field were counted and expressed as the relative percentage of total cells. (**C**) Cellular viability of mNSCs was measured on days 1, 3, and 5 after treatment in medium, except B-27, with WKYMVm at a concentration of 0.01–10 µM using Cell Counting Kit-8 (CCK-8) assay. Data indicate mean ± SD. * *p* < 0.05, ** *p* < 0.01, and *** *p* < 0.001 versus control (*n* = 10).

To explore whether WKYMVm promotes the proliferation of mNSCs, we evaluated the effect of WKYMVm on the proliferation of mNSCs by measuring Ki67 expression, which is expressed in proliferating cells. Treatment of mNSCs with 0.1 and 1 µM WKYMVm promoted the proliferation of mNSCs (Figure 5A,B). In addition, to further verify whether WKYMVm increases the survival of mNSCs, we investigated the effect of WKYMVm in a supplement-free medium that removed B-27. When WKYMVm was added to the culture conditions in the supplement-free medium, WKYMVm significantly increased the survival of NSCs at day 5 (Figure 5C). These results suggest that WKYMVm enhances the viability of mNSCs, which stimulates expansion, proliferation, and survival.

## 4. Discussion

Neural stem cells, in their self-renewal capacity, involve cellular responses such as migration and proliferation. Their differentiation potential generates major neuronal cell types such as neuronal and glial cell lineages [35,36]. Based on these characteristics, NSCs can be applied to the recovery of neurological diseases, including central nervous system disorders and degenerative neurodegenerative diseases such as Alzheimer’s disease, Parkinson’s disease, stroke, and drug screening to develop therapeutic agents for these diseases [37,38]. Therefore, it is fundamental to approach them based on the mechanism of adult-derived stem cells to apply NSCs in neurodegenerative diseases that are age-dependent due to the death of neurons. Moreover, the isolation and maintenance of NSCs from adults is important because differences in pathological characteristics, physiological functions, and molecular levels of NSCs can be directly analyzed according to neurological disease or disease progression [39,40]. Therefore, in this study, adult mNSCs were isolated from the subventricular zone of the brain. We demonstrated that adult-derived mNSCs exhibited phenotypes known as NSCs through two culture systems—neurospheres or adherent monolayers—and that adult-derived mNSCs differentiate into neurons, astrocytes, microglia, and oligodendrocytes, respectively.

Neural stem cells are an ideal cell therapy resource because they can regenerate damaged neuronal tissue and show potential to improve the therapeutic effects in transplantation of disease models [41,42,43]; however, they are limited in obtaining and expanding cells for autologous transplantation. Therefore, it is necessary to identify the factors that regulate and maintain the activity of endogenous neural stem cells. Growth factors such as brain-derived neurotrophic factor (BDNF), fibroblast growth factor 2 (FGF-2), epidermal growth factor (EGF), and insulin-like growth factor (IGF) play a crucial role in regulating and maintaining the function of NSCs [44,45,46]. The current study demonstrated that activation of FPR2 induced by WKYMVm stimulated chemotaxis migration and cell expansion of adult-derived mNSCs. Furthermore, incubation of adult-derived mNSCs with WKYMVm in a supplement-free medium improved survival. WKYMVm, identified by screening a peptide library, is a strong agonist of FPR2 [29]. Furthermore, WKYMVm induces endothelial cell migration, proliferation, and tube formation ability [47] and promotes neovascularization at the injury site by increasing the migration, homing, and mobilization of endothelial progenitor cells to peripheral blood in in vivo models such as hindlimb ischemia and myocardial infarction [48,49]. These studies show that WKYMVm is effective for tissue regeneration induced by the activation of endogenous stem or progenitor cells and supports our data on promoting the cellular response of mNSCs. In the future, it is necessary to investigate further the effect of WKYMVm on the regulation of NSC activity in vivo.

Although the stimulatory effect of WKYMVm on the cellular responses of NSCs has not been reported, the involvement of FPRs in migration and differentiation has been documented. The expression of FPRs is increased during neuronal differentiation of NSCs, and the activity of these receptors promotes neuronal differentiation [50]. Blockade of FPRs not only significantly inhibited FPR agonist-induced migration of NSCs in vitro and in vivo, but it also abrogated neuronal differentiation [30]. Formyl peptide receptor 2 is involved in accelerating senescence of hippocampal NSCs by amyloid-β_42_, a major component of amyloid plaque formation, a representative cause of Alzheimer’s disease (AD) [51]. In the AD model, annexin A1, an agonist of FPR2, stimulates phagocytosis of amyloid-β by microglia, increasing amyloid beta degradation and regulating the inflammatory response [52]. In addition, FPR2 function is regulated in the pro-inflammatory response, and treatment with FPR2 agonists stimulates the response of microglial cells to chemoattractant [53]. The expression of FPRs has a functional role in mesenchymal stem cells, suggesting a role for these receptors in stem cell migration and adhesion for tissue repair in damaged and inflammatory sites [54]. These reports suggest that FPR2 is functionally important not only in NCSs but also in regulating the cellular activity of several stem or progenitor cells and in a variety of cells exposed to specific environments.

The therapeutic candidates and technologies, including drugs and stem cells, to treat damaged brain and epilepsy and to restore nerve cells are being studied for clinical application [55,56,57]. Despite advances in cell therapy in regenerative medicine, stem cell-based replacement therapy has drawbacks such as its low cell yield, low transplantation rate, high costs, and low safety [58,59]. To overcome the clinical limitations of stem cell therapy, therapeutics targeting endogenous stem cells in vivo are promising. Therefore, for the therapy and recovery of the damaged brain, a clinical application that can treat the damaged brain through recruitment and neurogenesis of these cells, based on endogenous neural stem cells present in the neurogenic area, may be a useful approach [57]. In the present study, regulation of mNSCs by WKYMVm has been demonstrated in vitro; however, it is essential to elucidate the therapeutic potential of WKYMVm to repair damaged brains in vivo. Therefore, WKYMVm, which can solve the cost and safety issues associated with stem cell-based therapies, and its potential in regulating NSC activity in vivo need to be further explored in the treatment of models of brain diseases that require neurogenesis, such as brain injury or nervous system disorders.

In conclusion, our data show that the synthetic peptide WKYMVm, which activates FPR2, enhances the chemotaxis and viability of adult-derived mNSCs. These results suggest that WKYMVm may serve as a novel regulatory factor of NSCs and contribute to the application of NSCs for cell replacement therapy in neurological diseases in the field of regenerative medicine.

## Supplementary Materials

The following are available online at https://www.mdpi.com/article/10.3390/life11111248/s1, Figure S1: Original western blot images from Figure 2C. (A) Uncropped western blot images containing molecular weight markers of mNSCs and primary neurons using Nestin, NeuN and Actin are shown. (B) Ponceau S staining of membranes after protein transfer.
