# Supplementary material for: Stimulation of the Migration and Expansion of Adult Mouse Neural Stem Cells by the FPR2-Specific Peptide WKYMVm
Source: Life (Basel). 2021 Nov 17;11(11):1248. doi: 10.3390/life11111248 (PMC8622362; doi:10.3390/life11111248)
Supplement: Supplementary file 1 [file life-11-01248-s001.zip › life-1451273-supplementary.pdf]

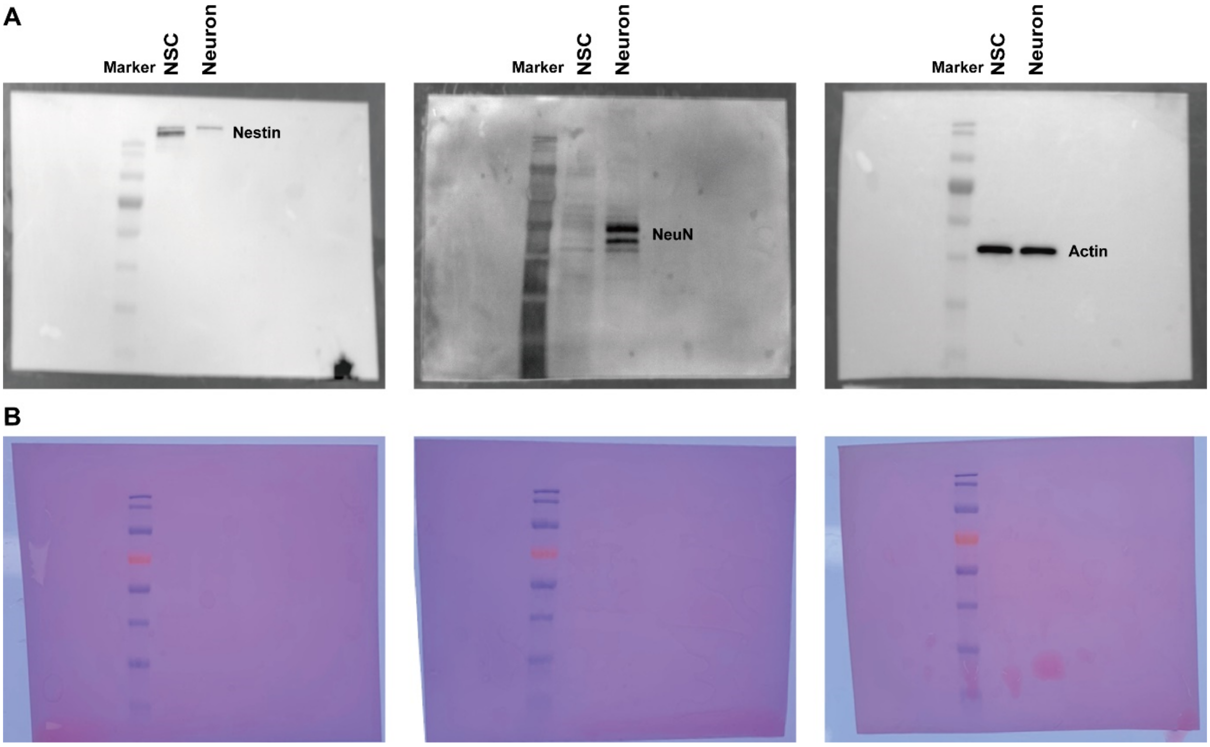

Supplementary Figure S1. Original western blot images from figure 2C. (A) Uncropped western blot images containing molecular weight markers of mNSCs and primary neurons using Nestin, NeuN and Actin are shown. (B) Ponceau S staining of membranes after protein transfer.
